# Assessing Cyanobacterial Harmful Algal Blooms as Risk Factors for Amyotrophic Lateral Sclerosis

**DOI:** 10.1007/s12640-017-9740-y

**Published:** 2017-10-03

**Authors:** Nathan Torbick, Beth Ziniti, Elijah Stommel, Ernst Linder, Angeline Andrew, Tracie Caller, Jim Haney, Walter Bradley, Patricia L. Henegan, Xun Shi

**Affiliations:** 1 Applied Geosolutions, 55 Main St, Suite 125, Newmarket, NH 03857 USA; 2 Department of Neurology, Dartmouth College, Hanover, NH USA; 3 Department of Mathematics and Statistics, University of New Hampshire, Durham, NH USA; 4 Dartmouth-Hitchcock Medical Center, Lebanon, NH USA; 5 Department of Biological Sciences, University of New Hampshire, Durham, NH USA; 6 Department of Neurology, University of Miami Miller School of Medicine, Miami, FL USA; 7 Department of Geography, Dartmouth College, Hanover, NH USA

**Keywords:** Amyotrophic lateral sclerosis, Cyanobacterial harmful algal blooms, BMAA, Remote sensing, Spatial epidemiology

## Abstract

Reoccurring seasonal cyanobacterial harmful algal blooms (CHABs) persist in many waters, and recent work has shown links between CHAB and elevated risk of amyotrophic lateral sclerosis (ALS). Quantifying the exposure levels of CHAB as a potential risk factor for ALS is complicated by human mobility, potential pathways, and data availability. In this work, we develop phycocyanin concentration (i.e., CHAB exposure) maps using satellite remote sensing across northern New England to assess relationships with ALS cases using a spatial epidemiological approach. Strategic semi-analytical regression models integrated Landsat and in situ observations to map phycocyanin concentration (PC) for all lakes greater than 8 ha (*n* = 4117) across the region. Then, systematic versions of a Bayesian Poisson Log-linear model were fit to assess the mapped PC as a risk factor for ALS while accounting for model uncertainty and modifiable area unit problems. The satellite remote sensing of PC had strong overall ability to map conditions (adj. R2, 0.86; RMSE, 11.92) and spatial variability across the region. PC tended to be positively associated with ALS risk with the level of significance depending on fixed model components. Meta-analysis shows that when average PC exposure is 100 μg/L, an all model average odds ratio is 1.48, meaning there is about a 48% increase in average ALS risk. This research generated the first regionally comprehensive map of PC for thousands of lakes and integrated robust spatial uncertainty. The outcomes support the hypothesis that cyanotoxins increase the risk of ALS, which helps our understanding of the etiology of ALS.

## Introduction

Concern over toxins and public health threats resulting from cyanobacterial harmful algal blooms (CHABs) have gained attention as reoccurring seasonal blooms persist in many waters. Cyanobacteria are particularly noxious when anthropogenic eutrophication (i.e., intensive agriculture, excess fertilizers, and runoff) of water bodies causes large concentrations of nutrients to produce massive cyanobacterial blooms. While blooms often cause local authorities to warn of acute health risks, the health impacts of chronic exposure to low or moderate levels of cyanotoxins are also largely unknown and potentially more pivotal for certain diseases and illnesses. Broadly, cyanotoxins can be described as having negative health impacts and can be grouped by the chemical structure, tissue, or target systems (e.g., neurotoxic, hepatotoxic, dermatotoxic). Cyanotoxins found within inland freshwater lakes include saxitoxins, anatoxin, cylindrospermopsins, lyngbyatoxins, hepatotoxins, and microcystins, which tend to be the most frequently reported by health agencies (Rao et al. 2002; Codd et al. 2005; Lévesque et al. 2014).

Cyanobacteria produce a variety of toxins that have human health implications, including beta-N-methyl-amino-L-alanine (BMAA) (Codd et al. 2005). The 100-fold higher incidence of ALS/parkinsonism-dementia complex (ALS/PDC) documented among the Chamorro people of Guam in the 1950s and 1960s implicated the cyanobacterial neurotoxin BMAA found in components of their diet (Mulder and Kurland 1987; Cox and Sacks 2002; Cox et al. 2003). BMAA has been demonstrated to be concentrated in the brains of ALS patients (but not controls) in Florida (Pablo et al. 2009) and to be mis-incorporated into neuronal proteins via the L-serine tRNA-synthetase system (Dunlop and Rodgers 2011; Dunlop et al. 2013). Two pathogenic mechanisms have recently been recognized both in sALS and familial cases: (1) protein misfolding (Mulligan and Chakrabartty 2013; Ogawa and Furukawa 2014; Grad and Cashman 2014; Ravits 2014), which is probably the major mechanism by which the cyanobacterial neurotoxin BMAA produces chronic neurotoxicity (Dunlop et al. 2013), and (2) impairment of RNA metabolism (Robberecht and Philips 2013). Recent findings show that chronic dietary exposure of vervets to BMAA caused neurofibrillary tangles and amyloid deposits similar to those in the brains of patients with ALS/PDC in Guam, supporting the notion of BMAA as an environmental trigger (Cox et al. 2016).

Evidence has shown linkages between water quality, cyanobacteria, and high ALS incidence (Caller et al. 2013; Torbick et al. 2014). Research has demonstrated the presence of BMAA in fish and crustaceans in the human food chain in Florida, Chesapeake Bay, Baltic Sea, France, and Sweden (Jonasson et al. 2010; Brand et al. 2010; Masseret et al. 2013; Mondo et al. 2012; Field et al. 2013). Clusters of ALS have been reported near cyanobacterial bloom outbreaks in France, Japan, New Hampshire, and Wisconsin (Sienko et al. 1990; Doi et al. 2010; Masseret et al. 2013; Caller et al. 2012). Caller et al. (2009) shows a statistically significant increased incidence of ALS in subjects residing within 0.5 miles of a New Hampshire lake that experienced cyanobacteria blooms. Torbick et al. (2014) showed increasing odds of belonging to an ALS hot spot with poorer water conditions that favor cyanobacteria in northern New England. Potential routes of exposure include aerosolization, dermal contact, ingestion of water, and dietary exposure through the aquatic food web (Banack et al. 2010, Stommel et al. 2013, Banack et al. 2015). However, measuring CHAB and toxins is very challenging given their dynamics and gene expression, the distribution of waterbodies across a landscape, and the tools required for precise measurements.

### Satellite Remote Sensing CHAB Exposure Mapping

The number, extent, and distribution of lakes in northern New England prohibit comprehensive assessment of cyanobacteria or toxins using traditional point sampling. The use of satellite remote sensing for mapping inland lakes conditions and CHABs has been well documented (e.g., Torbick et al. 2013; Torbick and Corbiere 2015a, b). Historically, CHAB or HAB algorithms have been developed for individual lakes and for specific sensors. Often, chlorophyll-a (chl-a) concentration, a measure of total phytoplankton biomass, are retrieved as a surrogate for HAB exposure. However, proxies such as phycocyanin concentration (PC) (μg/L), population density (cells/L), or cyanobacterial biovolume (μm3/L), which are more sensitive to pigments of cyanobacterial species or relative amount, have also been developed using satellite data. For example, Vincent et al. (2004) used Landsat 5 TM and ETM+ 7 to map a phycocyanin concentration index highlighting *Microcystis* for two time periods in the western basin of Lake Erie. In an evaluation of transferability, Lunetta et al. (2015) evaluated the use of a MERIS-derived Cyanobacteria Index, which had originally been developed for Lake Erie (Wynne et al. 2008; Stumpf et al. 2012), to lakes in eastern USA and found moderate success for low and high levels of cyanobacteria biovolume concentrations. The use of PC further improves upon Torbick et al. (2014)’s analysis of the effect of water quality on ALS risk since PC is a more direct measure of cyanobacteria concentration (Stumpf et al. 2016) in northern New England waters and hence is a more specific indicator of cyanotoxin-producing cyanobacteria than total algal biovolume or chl-a metrics.

Typically, tradeoffs are required between satellite resolutions (spatial, temporal, and spectral) as well as cost and availability. These also influence algorithm approaches as some sensors are more capable of diagnostic retrieval (analytical) rather than statistical algorithms although many approaches today blend techniques depending on objectives (Mouw et al. 2015). Given the size, shape, and distribution of inland lakes, moderate resolution platforms such as Landsat-8 are required for wall-to-wall or total mapping of lakes across a region. For example, Torbick et al. (2013) used Landsat band ratio regression models when mapping a suite of comprehensive indicators across a dozen Landsat scenes (designated by path/row grid) required to cover Michigan. The broad spectral channels of Landsat prohibit easily scalable analytical algorithms that are useful across lake types or conditions over a large region. Techniques leveraging valuable application-specific wavelengths on sensors such as MERIS or MODIS (i.e., Simis et al. 2005; Wynne et al. 2008; Stumpf et al. 2012) are not capable of mapping small- to medium-sized lakes given the spatial resolution of these platforms. Further, while research has shown advantages of narrow band hyperspectral data for mapping cyanobacteria concentration or phytoplankton functional types (e.g., Gietelson et al. 2000; Simis et al. 2005; Hunter et al. 2010), there is no cost-effective or operational satellite platform capable of comprehensively mapping lakes over a large region with narrow spectral channels.

### Objectives

The objective of this work was to develop cyanobacterial harmful algal blooms (CHAB) exposure maps using satellite remote sensing calibrated with in situ observations to assess CHAB exposure as a potential risk factor for ALS considering scales and spatial uncertainty.

## Methods

### Study Site

Northern New England, USA (NNE; Maine, New Hampshire, Vermont), is a diverse socio-ecological system representing a range of landscapes, populations, and lakes. A history of settlement, farming, and timber industry segmented the landscape during the past two centuries. Natural habitat is broadly classified as Eastern Temperate Forest with level 3 ecoregions of Atlantic Maritime Highlands, Northeastern Coastal Zone, and Acadian Plains and Hills (Omernik 1987). The interior of NNE has a humid continental climate (Dfb: Köppen climate classification) with cold winters and seasonal patterns. NNE has a total area of 140,786 km^2^ with 9060km^2^ of lake surface area and a population of 3,283,562 million ranging from small villages to large cities. Over the past four decades, urban sprawl and impervious surfaces (i.e., conversion of natural land covers to man-made surfaces such as pavement) have increased the most in coastal and interior NNE relative to New England with much of the development surrounding lakes and lake communities (Torbick and Corbiere 2015a, b). Recent work has shown lake temperatures are increasing at a rate of 0.8 °C/decade in the region (Torbick et al. 2016), which will likely increase the frequency, duration, and magnitude of CHAB events. There are 4117 waterbodies greater than 8 ha and generally lake water quality is considered “good” in NNE with 82% categorized as oligotrophic and mesotrophic according to Landsat derived Trophic Status Index maps (Torbick et al. 2014).

### Human Health Case Data

Our team has been building ALS case data in multiple regions, including NNE, for the past 10 years. The database used in this analysis included date of birth, sex, and residential longitude/latitude coordinates for cases collected between January 1999 and October 2009 similar to that used by Caller et al. (2013) and Torbick et al. (2014). Records from Dartmouth Hitchcock Medical Center (DHMC), the Muscular Dystrophy Association of Northern New England, regional clinics, and surveys were searched to identify cases of ALS diagnosed with dates. When possible, we confirmed accuracy of diagnosis, year of diagnosis, demographic history of patients identified by review of medical records, the Social Security Death Index, obituaries, and data supplemented from questionnaires. Nine cases only had a town name with no coordinates. These cases were assigned town centroid spatial location using the geocode function in the R package ggmap, which makes use of Google Maps (Kahle and Wickham 2013). This procedure did add spatial uncertainty for distances less than the town aggregation level for these few patients. Furthermore, the spatial extent for this database was restricted to the states of Vermont and New Hampshire and excluded the counties of Bennington (VT) and Cheshire, Hillsborough, Rockingham, and Strafford (NH), giving a total of 347 (in this selected region) ALS cases. This sub-region of NNE was selected since the ALS dataset being used in this analysis is suspected to underestimate the risk for the entire NNE region as it is likely that portions of the NNE population travel to other urban area medical centers (e.g., Boston) and thus are not within the clinic/hospital catchments of our dataset (Caller et al. 2015).

### In Situ Lake Measurements

**Fig. 1:**
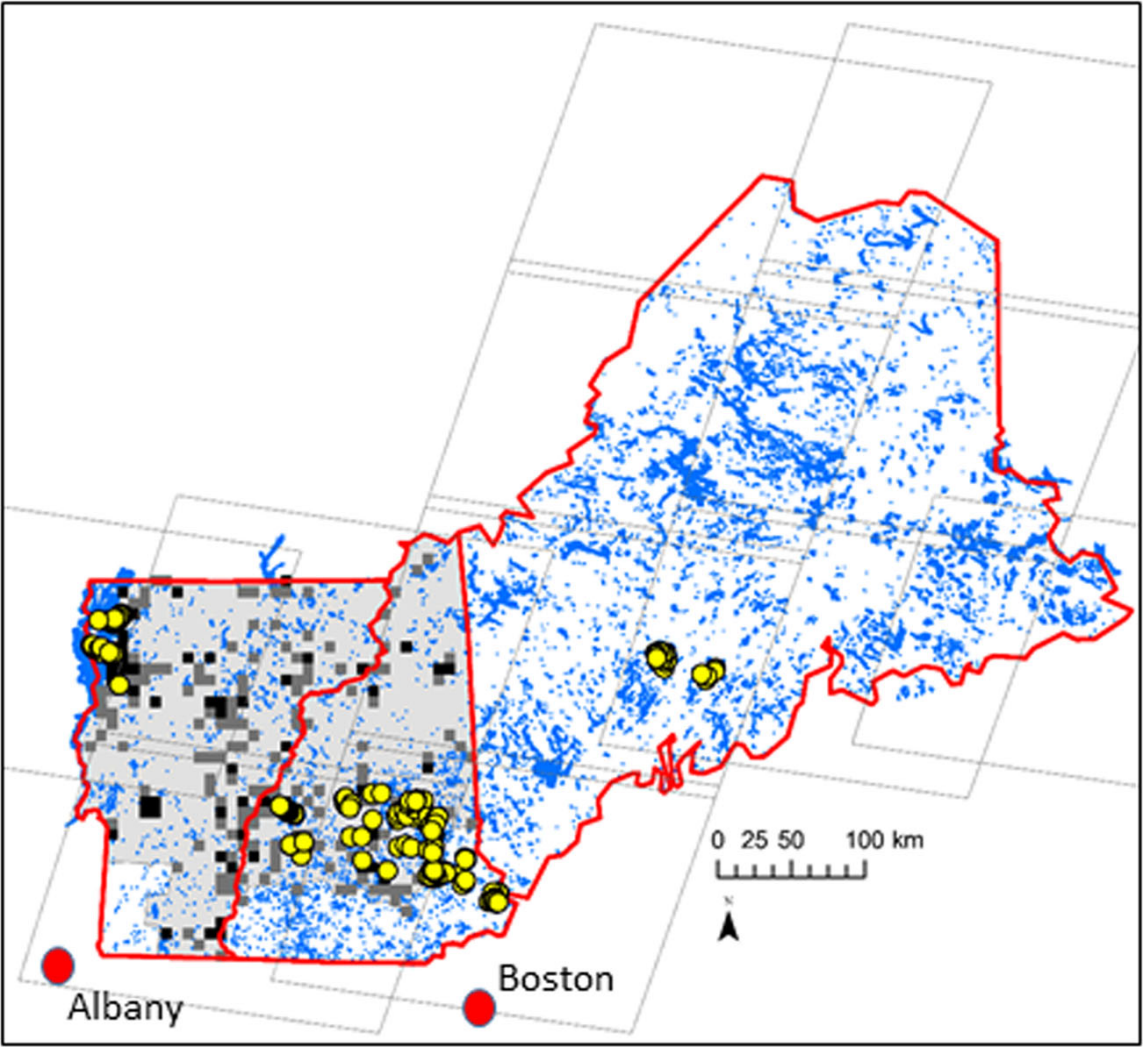
Northern New England study region showing lakes (*blue*), Landsat path row tile footprints, gridded ALS case data aggregated to 8 km units for privacy, boundary extent (*gray*), and lakes with in situ data collection (*yellow*)

A field campaign to collect near simultaneous (in regard to satellite overpass) in situ measurements of phycocyanin concentration and other parameters across the region was carried out during the summers 2014, 2015, and 2016 (Fig. 1). The campaign was coordinated with government agencies (EPA, New Hampshire Department of Environmental Services, Maine Department of Environmental Protection, Vermont Department of Environmental Conservation) and university labs to ensure cross calibration and efficiency. A stratified lake sampling approach was executed that considered size, trophic status, depth, access, path row (location), watershed, and practical logistic factors (e.g., safety, drive time). Target satellite overpasses (path row) were coordinated with strategic lakes while considering local weather patterns (clouds, wind, humidity) during overpasses in an attempt to obtain a high number of diverse and robust samples under quality (clear sky) conditions. At each lake, local conditions were assessed and a sample location representative of an approximate 3 × 3 Landsat pixel array was pursued to allow for linkage between the in situ and satellite remote sensing. Medium and larger lakes (>200 ha) with spatial variability had multiple samples from different bays, “open” water, or noteworthy locations (e.g., near damn). A total of 305 unique observations from 79 different waterbodies across Maine, New Hampshire, and Vermont were obtained during July, August, and September. Conditions ranged from a small, 8.1-ha hypereutrophic pond (Showell Pond) to a large 127,000-ha lake (Champlain) with varying CHAB conditions across bays.

A well-calibrated YSI EXO-1 multi-parameter sonde was used to measure in situ cyanobacteria concentrations along with measurements of chlorophyll a, dissolved oxygen, fluorescent dissolved organic matter (FDOM) as a surrogate for chromophoric (colored) dissolved organic matter (CDOM), and a suite of other parameters (e.g., secchi depth, temperature, total dissolved solids, conductivity). Instrument measurements focused on 30–50 cm depth or near surface while periodic epilimnion, integrated tube, and vertical profiles were also collected. The “cyanobacteria” sensor measures phycocyanin pigments using in vivo fluorometry (IVF) in real time, detecting concentrations with a resolution of 1 cell/mL (0.1 relative fluorescence units/RFU). The instruments were calibrated and cross compared against extracted concentrations, standards, and other probes before sampling began each season, within season, and after season to correct for any potential drift. Since PC/cell can be variable depending on culture conditions, we also cross calibrate on PC pigment while also assessing against cultured *Microcystis*. Periodic integrated tube samples, plankton tows, and enzyme-linked immunosorbent assays (ELISA) were executed to gauge vertical profile structure, enumerate taxa, assess toxicity at a subset of lakes, and ensure cross calibration between in situ and probe instruments. The sonde was ported to a handheld integration device to simultaneously record Global Positioning System data and instrument observations. A StellarNet Inc. bluewave® spectroradiometer was used to collect periodic in situ radiometric measurements following best practices (e.g., Torbick and Becker 2009). The handheld hyperspectral device measures the 350–1150 nm range using a 16-bit digitizer and holographic diffraction grating (600 g/mm) CCD with a signal-to-noise ratio of 1000-to-1. The handheld hyperspectral measurements were used to help gauge conditions, spectral absorption characteristics, and qualitatively support preprocessing decisions.

### Satellite Remote Sensing Mapping

A multi-year (see below) collection of in situ measurements was executed across 79 lakes of varying size and conditions, multiple path rows, and multiple time windows targeting Landsat 7 Enhanced Thematic Mapper Plus (ETM+) and Landsat 8 Operational Land Imager (OLI) overpasses. Landsat follows a Sun-synchronous orbit at an altitude of 705 km with a 16-day repeat window; each Landsat satellite (7 and 8) being offset to provide 8-day overpass repeats for a given foot print. These platforms capture observations in the visible (vis/0.45–69 μm), near-infrared (nir/0.75–0.90 μm), and shortwave-infrared (swir/1.55–1.75, 2.08–2.35 μm) at 30 m spatial resolution. Data were obtained as L1T from Earth Explorer with standard radiometric and geometric terrain corrections.

For inland lake mapping, a tradeoff is required considering number of samples, timing of overpass, atmosphere and weather conditions, and dynamics of CHABs. Longer temporal windows between in situ sampling and overpass provide more samples for modeling; however, potentially longer intervals present greater uncertainty concerning the stability of the conditions. This research followed precedence (i.e., Torbick et al. 2013; Lunetta et al. 2015) and had +/−2-day window. This resulted in 6 unique dates from 2014 (i.e., Days Of Year 237, 238, 245, 246, 251, 260), 4 unique dates from 2015, and 4 from 2016. Preprocessing routines for atmospheric correction built upon previous efforts. In summary, the atmospheric correction routines tested included the use of MODIS Aerosol Optical Depth (Level 3 MOD08_D3) to drive the Second Simulation of the Satellite Signal in the Solar Spectrum (6S) radiative transfer model (Vermote et al. 1997) to generate water-leaving radiance and surface reflectance measurements (Ledaps_m_). We also compared these outcomes to preprocessing routines that followed Vanhellemont and Ruddick (2014) and Vanhellemont and Ruddick (2015) to generate water-leaving radiance (rhow) and reflectance (rhoam) measurements by using a SWIR-based correction approach to adjust for Rayleigh and aerosol scattering. Correcting for atmosphere, while challenging, has advantages for transferability and more robust mapping models. For clouds and shadows, Function of Mask (Zhu and Woodcock 2012), Automated Cloud Classification Algorithm (Masek et al. 2006), and Band Quality Assessment (BQA) were applied using Ledaps and L8SR, as required for ETM+ and OLI. Any cloud or shadow pixels, along with Scan Line Corrector (SLC) gaps, were treated as no data.

A spatiotemporal database was built linking the Landsat overpasses to the in situ measurements. Sampling points were buffered to represent a 3 × 3 Landsat pixel array (visible bands; 3 × 3 pixels = 90 m × 90 m). These sampling units were then intersected with an inward buffered lake vector boundary to ensure no coastline or mixed pixel problems. The mean value for these areas was used as this helps capture potential variability of positioning error in either the georegistration or sample location. Strategic semi-analytical regression models were examined using variables shown to have spectral relationships with inherent optical properties in previous studies. Strategic independent variables (i.e., bands and ratios) were systematically added and removed while examining statistical performance and residuals. Withheld, out-of-sample adjusted *R* ^2^, significance values, root mean square error (RMSE), and Akaike Information Criterion (AIC) were used to assess performance. The result of the satellite mapping was a map of phycocyanin concentration (i.e., CHAB exposure) for all lakes greater than 8 ha in northern New England.

### Statistical Modeling

There are several challenges in assessing the relationship between phycocyanin concentrations and ALS risk that go beyond the challenges in mapping it. For one, it is difficult to know exactly to what extent people are exposed to specific lakes. Thus, a common approach is to compare case residence locations to average exposures based on a certain proximity scale (Waller and Gotway 2004; Torbick et al. 2014). In a statistical framework, this comparison takes the form of a generalized linear regression model, which for our study is a Poisson regression that compares exposure levels to case counts adjusted for the density and demographic differences in the background population at risk (Diggle et al. 1998).

Ongoing deliberation within the science community exists on the model specifications that should be used for Poisson regressions of environmental public health data. On the one hand, a correct modeling approach needs to account for spatial dependence in the data; however, on the other hand, the accounting for spatial autocorrelation can be computationally prohibitive depending on the exact form and size of the data (Banerjee et al. 2004), and the addition of spatial random effects can create variance inflation in the exposure effect parameter, meaning significant exposures may appear insignificant (Reich et al. 2006; Hughes and Haran 2013; Hughes 2015). Furthermore, model outcomes depend on the shape and size of modeling units (Wall 2004; Waagepetersen 2004; Li et al. 2012), the choice of the background population dataset (Tatem et al. 2011), and the proximity scale chosen to average exposure estimates (Torbick et al. 2014). To address these challenges, we use Bayesian inference estimated using Integrated Nested Laplace Approximation (INLA) as implemented in the R package INLA (Rue et al. 2009) for 64 different Poisson log-linear models that vary each of the following components: the use of spatial random effects (two choices), the size of the geographic modeling units (two resolutions), the background population dataset (two choices), and the proximity scale for PC concentration exposure (eight scales). This modeling approach addresses all the potential complications mentioned above in a robust and transparent manner.

Half the models we executed contain no spatial random effects, and the other half include the spatial random effects proposed by Besag et al. (1991), which are a convolution of independent and spatially correlated random effects, where the spatially correlated random effects are an intrinsic conditional autoregression. The Poisson log-linear model with BYM random effects is a special case of a Log-Gaussian Cox Process (LGCP) (Møller et al. 1998) and is commonly used when data come in the form of case counts on areal units defined by administrative areas. One advantage of this specification is the savings in computational costs for Bayesian inference since a numerical approximation known as INLA can be used for very fast estimation of the posterior marginal distributions (Rue et al. 2009). However, since administrative areas are often not related to the disease in question and may vary greatly in size and shape, this model specification has been criticized for poor and/or unexpected results (Wall 2004; Li et al. 2012).

Since our health data are available as point locations representing the residence of a disease case, we can avoid the negative effects by defining custom areal units similar in size, shape, and related to the disease. A regular lattice is one such set of custom areal units used (Li et al. 2012; Illian et al. 2012; Diggle et al. 2013) for which Waagepetersen (2004) has shown that when pixel sizes tend to zero, the approximate posterior expectations of the LGCP converge to exact posterior expectations. This means that the regular lattice provides an approximate continuous specification of disease intensity, which has the potential to incorporate data on environmental risk factors that are available at high spatial resolutions (Diggle et al. 2013). Despite the approximate continuous specification, a specific discretization is subject to the ecological fallacy (Waller and Gotway 2004; Diggle et al. 2013). Thus, using the regular lattice as our geographic modeling units, we compare two resolutions, a 4-km resolution and an 8-km resolution, which were chosen to balance computational efficiency with disease process spatial variability. Specifically, the 8-km resolution was chosen to account for spatial uncertainty in some case locations where only a town name was available and because the median square area of these towns was 66.3 km^2^. The 4-km resolution was chosen following a procedure outlined by Diggle et al. (2013) where a preliminary estimate of the disease process spatial variability was obtained via minimum contrast.

**Fig. 2:**
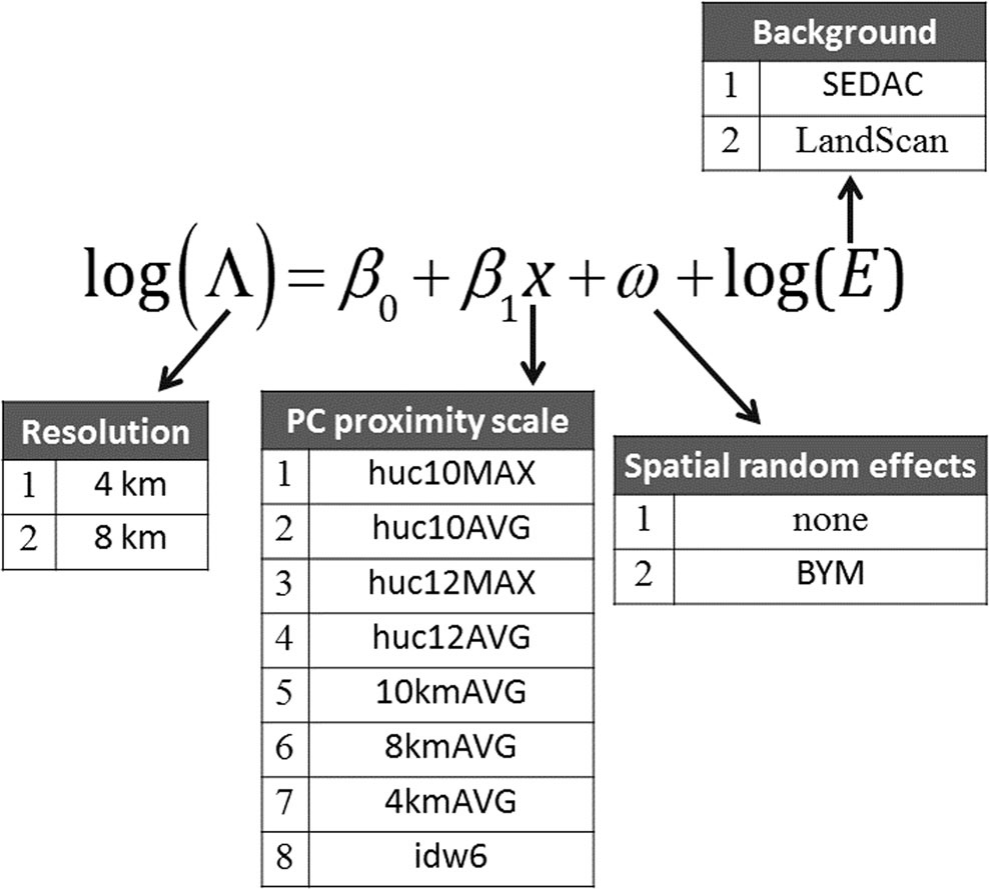
Breakdown of modeling components considered in this study resulting in 64 unique models

Adjusting the case counts in the 4 and 8 km regular grids for the density and demographic differences in the background population at risk is done by calculating two sets of expected counts based on gridded population products representing the region’s population at the year 2000, one provided by the Socioeconomic Data and Applications Center (SEDAC) and the other a product of OakRidge National Laboratory (LandScan 2000). Expected counts are then used as a fixed offset parameter in the Poisson log-linear models (see log(*E*) in Fig. 2). Expected counts were indirectly and internally standardized, which means they represent the number of the cases expected in a specific pixel location assuming the population in that pixel location contracts ALS at the same rate as an internal standard population (Waller and Gotway 2004, chapter 2). Rates are age and sex specific following the age/sex classes defined in Noonan et al. (2005), and the internal standard population is the superpopulation containing all pixels within the study spatial extent. The age/sex specific rates were calculated as follows:

$$ {r}_i=\frac{\sum_{\mathrm{all}\ x}{O}_i(x)}{\sum_{\mathrm{all}\ x}{n}_i(x)}, $$

where *i* = 1 , 2 ,  …  , 12 is the identifier for one of the 12 age/sex classes defined by Noonan et al. (2005), *x* represents a pixel location, *O*_*i*_(*x*) is the number of observed ALS cases in the pixel *x* having age/sex class *i*, and *n*_*i*_(*x*) is the population at risk in pixel *x* having age/sex class *i*. Following the calculation of the age/sex specific rates, the standardized expected counts for each pixel *x* are calculated as:

$$ E(x)=\sum_{\mathrm{all}\ i}{n}_i(x)\times {r}_i. $$

The *n*_*i*_(*x*) for the both *r*_*i*_ and *E*(*x*) are either the populations counts from SEDAC or from Landscan 2000. A square pixel *x* is either sized 4 or 8 km.

For PC proximity scales, we consider a suite of lake area-weighted averages and maximums for a range of distances from the centroid of the modeling unit (4 or 8 km square pixel) and for watershed scales in which the modeling unit centroids fall. Table 1 gives the names and details for each scale used in our multi-scale analysis evaluating PC exposures.

**Table 1:** Proximity scales of phycocyanin concentration (μg/L) used in the ALS modeling study

PC proximity scale	Description
huc10MAX	Maximum of lake averages within the Hydrological Unit Code 12 (HUC10) boundary assigned to 4(8)km lattice cells if cell centroid also falls within the HUC10 boundary
huc10AVG	Mean of lake averages within the HUC10 boundary assigned to 4(8)km lattice cells if cell centroid also falls within the HUC10 boundary
huc12MAX	Maximum of lake averages within the Hydrological Unit Code 12 (HUC12) boundary assigned to 4(8)km lattice cells if cell centroid also falls within the HUC12 boundary
huc12AVG	Mean of lake averages within the HUC12 boundary assigned to 4(8)km lattice cells if cell centroid also falls within the HUC12 boundary
4kmAVG	Mean PC of all 30 m lake pixels within a 4-km radius of 4(8)km lattice cell; when no lake intersects this radius, a value of 0 is assigned
8kmAVG	Mean PC of all 30 m lake pixels within an 8-km radius of 4(8)km lattice cell; when no lake intersects this radius, a value of 0 is assigned
10kmAVG	Mean PC of all 30 m lake pixels within a 10-km radius of 4(8)km lattice cell; when no lake intersects this radius, a value of 0 is assigned
idw6	Inverse distance weighted mean PC of 30 m lake pixel centroids to each 4(8)km lattice cell centroid, where 30 m cell centroids greater than a distance of 50 km were not included. Weights were $\frac{1}{{\mathrm{distance}}^6}$

The outcome of this modeling analysis is thus twofold. First, we seek to quantify the relationship between each of the spatial proximity scales of PC and ALS risk as well as compare differences in the scales. Second, we seek to quantify the impact of the fixed model components, i.e., the choice of background population, the choice of grid size, and the use of spatial random effects, on the estimate of the PC proximity metric’s effect on ALS risk and on the model’s fit as measured by the deviance information criterion (DIC) (Spiegelhalter et al. 2002) to ensure robust statistical analyses and address uncertainty.

## Results and Discussion

### Satellite CHAB Exposure Mapping

**Fig. 3:**
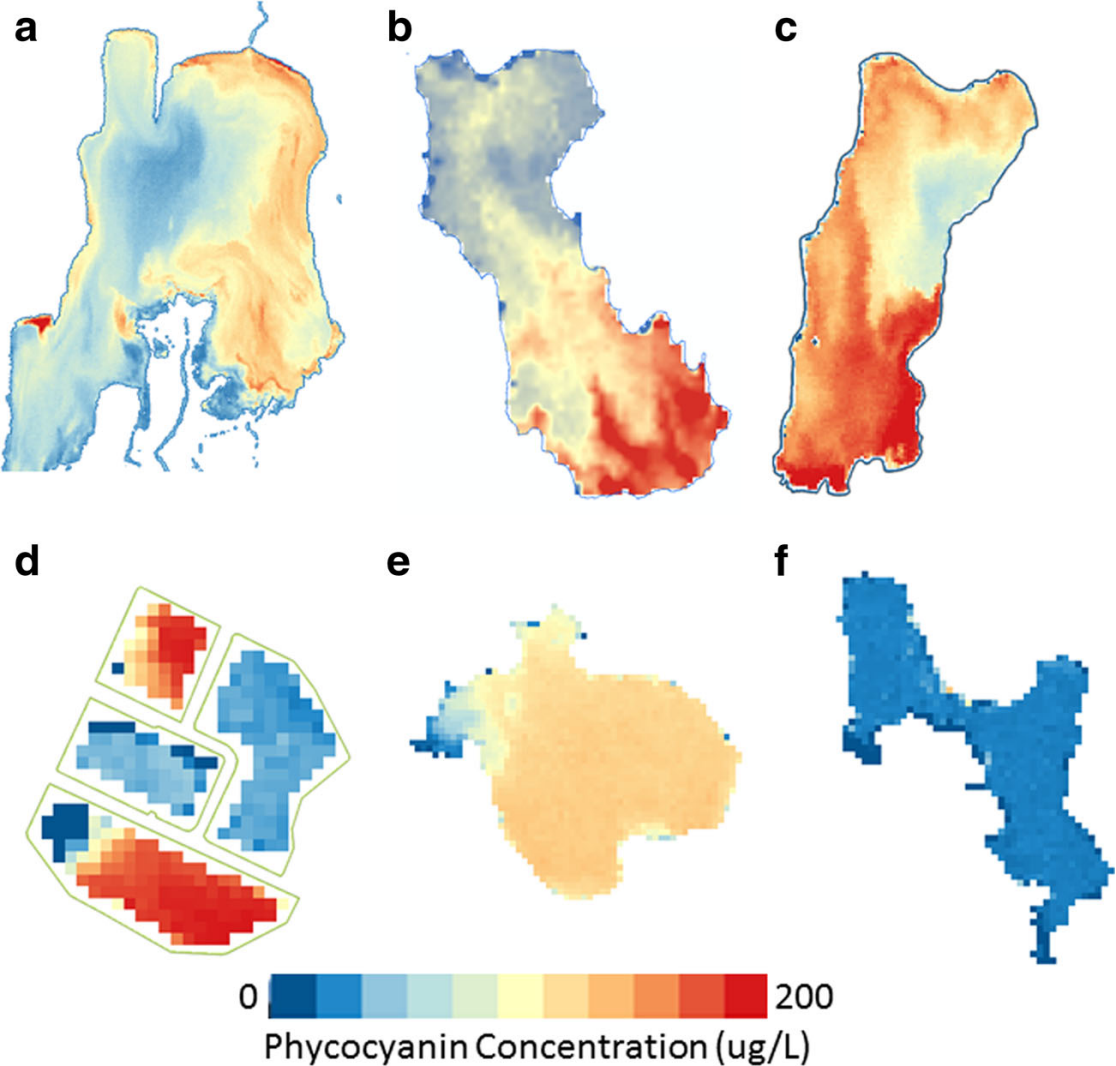
Landsat-derived maps of phycocyanin concentration for **a** Missisquoi Bay, Lake Champlain, **b** Shelburne Pond, **c** Lake Carmi, **d** South Sanford retention ponds, **e** Lake Attitash, and **f** Wenham Pond

Lake water estimates of CHABs and their toxins are severely limited in most regions. This research application represents the first regionally comprehensive map of PC for thousands of lakes. Figure 3 illustrates example waterbodies and highlights ability to capture within lake spatial variability of PC. The majority of lakes in NNE had low prevalence of CHAB during the overpass dates (circa 2015) used for this regional exposure map. Many lakes in NNE had no to low (<10 μg/L) PC for the dates observed. The number of lakes at example PC thresholds in NNE is shown in Table 2 while the mean lake PC for this time period was 7.4 μg/L (Table 2). A total of 415 lakes with NNE had a mean PC greater than 10 μg/L with 32 having a mean PC greater than 100 (μg/L). Watersheds (at the HUC12 scale) in northwest Vermont, southern New Hampshire, and Central Maine had hot spots of elevated mean PC at the watershed scale. A total of 37 watersheds have mean PC greater than 25 μg/L. Watersheds with very elevated PC (>100 μg/L) include Putnam Creek, (VT/NY), Mississquoi Bay (VT), Putnam Creek (NY/VT), Cathance and Androscoggin River (ME), Winooski River (VT), Riviere aux Brochets (Canada), Big Presque Isle stream (ME), and South Bay Lake Champlain (NY). Approximately 5% (*n* = 208) of all lakes >8 ha across NNE (*n* = 4117) had a maximum PC value >100 μg/L.

**Table 2:** Matrix of Landsat-derived mean lake phycocyanin concentration (μg/L) across the study region

	Mean	Lake	PC (μg/L)
State	>10	>50	>100
ME	284	56	16
NH	79	12	9
VT	52	18	7

The multi-season and multi-year in situ sampling campaign was combined with Landsat observations to generate exposure maps across northern New England. The semi-analytical algorithm (NIR/green + NIR/blue band ratio regression) calibrated with the in situ had a strong overall ability (withheld sample adj. *R* ^2^ 0.86; RMSE 11.92) to map PC. Both the Ledaps_m_ and rhow atmospheric corrections generated comparable PC distributions across the region, although using different coefficients. Since semi-analytical models were applied, the highest accuracy and most precise (RMSE, F-stat, adj. *R* ^2^) model was chosen as the CHAB exposure map, which well represented the CHAB conditions (at this snapshot in time) and spatial variability across the entire region. The use of moderate and freely available sensors (i.e., Landsat-8, Sentinel-2) is important as there are many small lakes and ponds across landscapes that act as potential exposure sources for populations.

Currently, multiple initiatives by organizations, such as the World Health Organization, the US Environmental Protection Agency, and US Geological Survey, are developing guidelines for classifying toxins in waters, health criteria, and decision making. Variations exist across drinking and recreational waters, and these are generally geared at acute health impacts and operational decisions such as drinking water treatment protocol. In the USA, some individual states also have guidelines and varying criteria such as the State of Vermont and their tiered lake sampling program based on cell counts and toxin analysis tied to management protocol such as posting signage on a public beach. Guidelines across these initiatives to date have been geared at observations of microcystins, chlorophyll-a concentrations, cyanobacteria biovolume, visible scums, foul odor, liquid chromatography mass spectrometry (LCMS), or ELISA tests for anatoxins and saxitoxins, and other metrics tuned to CHAB exposure.

No current PC guidelines or thresholds exist at local, state, or Federal levels or by the World Health Organization. Several initiatives are developing criteria based on combinations of toxicity, cell abundance, and measurements of algal biomass; however, no operational policies are in place at this time. Potentially, PC maps derived from satellite remote sensing can help inform guidelines and risk management decisions. The use of PC or chl-a would allow satellite remote sensing to play a strong role in supporting monitoring, reporting, and verification for CHAB public health programs. Currently, satellite remote sensing cannot directly detect toxins such as BMAA or microcystins; therefore, use of PC or other exposure metrics that can be accurately derived from satellite remote sensing can be considered. Potentially, approaches that model relationships between PC or chl-a parameters and toxins can be used; however, this remains an active research area and more effort is needed to scale up to a landscape-level assessment. The cost effectiveness, availability, large area coverage, and repeat frequency of remote sensing can support regional CHAB mapping programs to complement traditional sampling or scale-up studies to larger areas. This work showed robust PC mapping across multiple years, multiple path rows, and several sampling dates across a range of lake conditions, indicating usefulness in supporting public health investigations and CHAB tracking initiatives.

### Relationship of Water Quality to ALS Risk

**Fig. 4:**
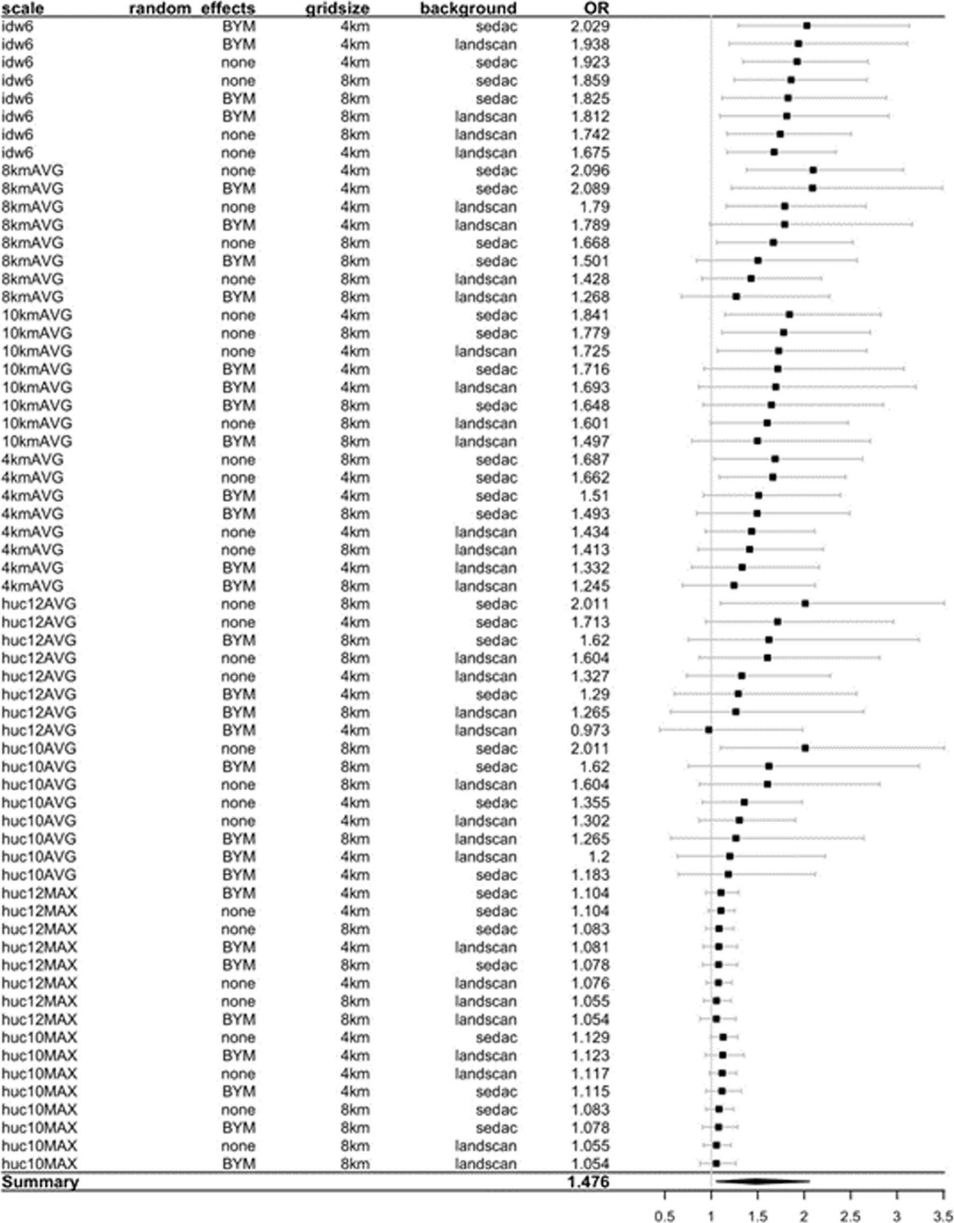
Odds ratios for each 1 μg/L increase in PC exposure for all models. *Left* is the estimated mean for each model and *middle* and *right* are the 95% confidence bounds for each model

We used a spatial epidemiological approach to evaluate the mapped PC exposure at various proximity scales as a risk factor for ALS, and multiple versions of the Bayesian Poisson Log-linear model were fit using INLA to account for uncertainty due to model choice. All the 64 models except for one had an estimated odds ratio greater than one, meaning that on average higher amounts of PC exposure are associated with higher risk of ALS in Vermont and New Hampshire (Fig. 4). However, lower 95% confidence bounds for all these models are not greater than 1. Furthermore, whether the lower bound is greater than 1 and the width of the intervals vary by PC proximity scale.

Significance of the PC exposure was determined using percentiles of the estimated effects’ marginal posterior distributions to calculate approximate *p* values, where for example, if the smallest positive percentile was the fifth percentile, then the calculated *p* value was 2 × 0.05 = 0.1. These are approximate since only the percentiles, 0.005, 0.025, 0.05, 0.1, 0.2, …, 0.9, 0.95, 0.975, and 0.995 were calculated from the estimated effects’ marginal posterior distribution. Thus, the smallest approximate *p* value of any of the models is 0.01. The distribution of approximate *p* values for all 64 model is shown in Table 3. Since multiple models are fit, one would expect a uniform distribution of *p* values if the phycocyanin concentration had no effect. However, there are more small *p* values than large *p* values (Table 3), which supports the hypothesis that PC exposure is a risk factor for ALS. This is consistent with the results of Torbick et al. (2014), which showed poorer water quality is associated with ALS hotspot membership, and further improves on these results by explicitly accounting for model uncertainty.

**Table 3:** Distribution of discretized *p* values for the statistical significance of the relationship between phycocyanin concentration (μg/L) and ALS risk from all 64 models

Distribution of *p* values		
Approximate *p* value	Count	Proportion
0.01	8	0.125
0.05	11	0.172
0.1	8	0.125
0.2	13	0.203
0.4	12	0.188
0.6	11	0.172
0.8	0	0
1	1	0.016
Total	64	1

**Fig. 5:**
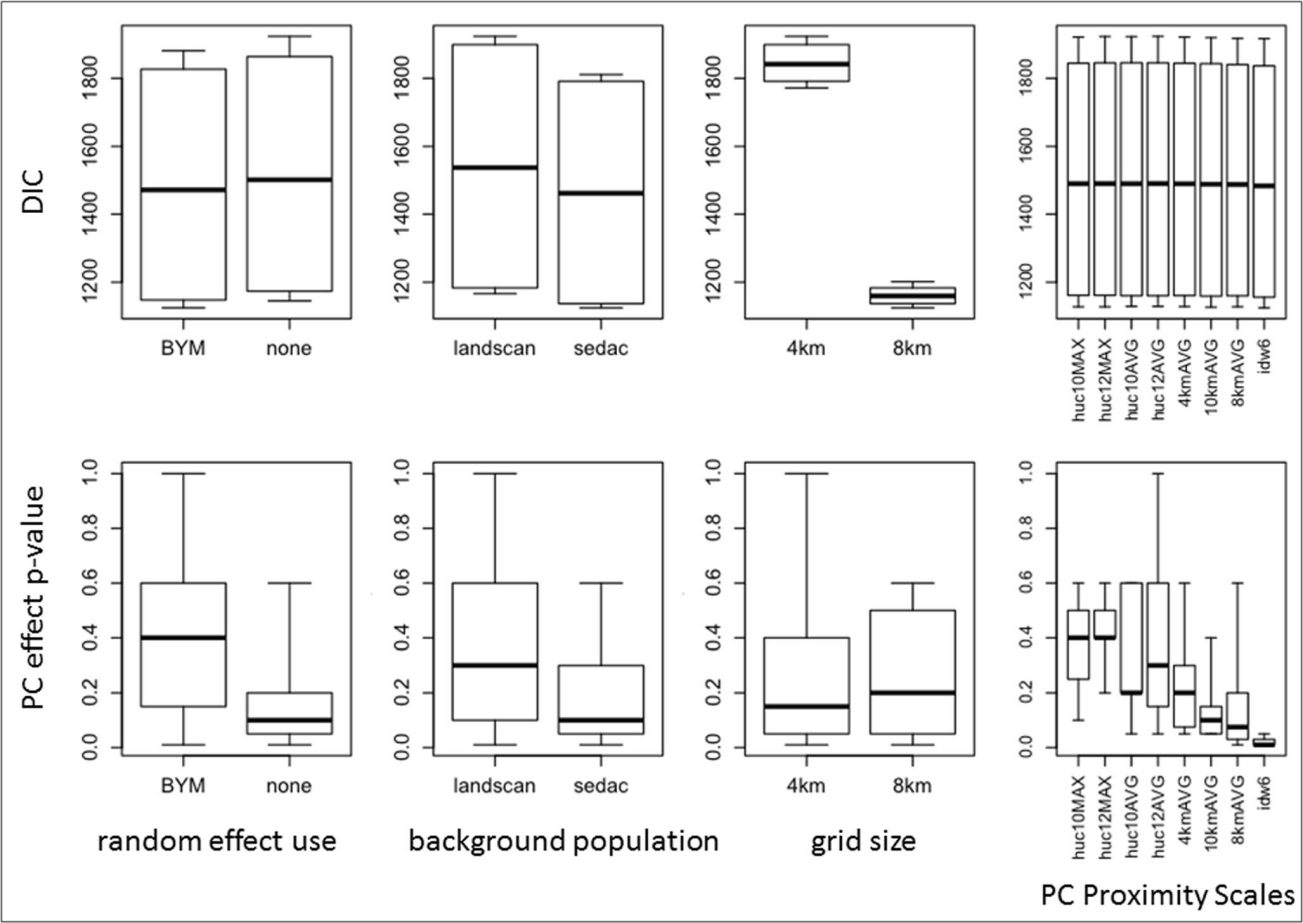
Boxplots comparing impact of model choices. For each of the model components, random effect use, background population, and grid size, there are 2 choices with 32 models for each choice. For the choice of PC proximity scale, there are 8 choices each with 8 models. *Top* boxplots compare DIC (deviance information criterion). *Bottom* boxplots compare *p* values of the effect of PC exposure

The impacts of differences in the background population datasets, the grid sizes, and the use of spatial random effects are measured in two ways; one by comparing the deviance information criterion (DIC) (Spiegelhalter et al. 2002) values where smaller DIC values indicate models that better explain the spatial variability of ALS risk, and two by comparing the *p* values of the PC exposure effect on ALS risk where smaller *p* values indicated a more significant effect. In terms of the model fit, the choice of grid size has the largest impact since this choice shows the greatest difference between DIC values (Fig. 5 top). Models with the larger grid size (8 km) have smaller DIC values (better fits), which may be due to better stability of population estimates in the rural regions and/or due to spatial uncertainty in exposure location. In terms of the significance of the relationship between the PC exposure and the spatial variability of ALS risk, there is a noticeable difference between the use and non-use of spatial random effects with smaller *p* values observed for non-spatial models (Fig. 5 bottom). From a model selection point of view, the differences in *p* values between spatial and non-spatial models is not particularly useful as neither the BYM random effects nor the non-spatial models are ideal (Reich et al. 2006; Hughes and Haran 2013; Hughes 2015). However, concordance across these models provides support for the identified relationships.

To investigate differences in the PC proximity scales, we compared DIC (Fig. 5 top right) and approximate *p* values (Fig. 5 bottom right). In terms of DIC (model fit), there is little difference between PC proximity scale choices when considering the eight models for each proximity scale varying random effect use, background population choice, and grid size. However, in terms of *p* values, the proximity scales 10kmAVG and idw6 that included farther distances had smaller *p* values, and all 8 models with the idw6 scale had *p* values of 0.05 or less despite the differences in use of random effects, choice of background population, and grid size. It could be that the inclusion of larger distances in these scales can better capture the population mobility in this predominately rural and suburban region. For example, the idw6 scale included distances representing a 30- to 40-min drive. However, this scale differs from the other averages in that it is an inversely weighted distance average where farther lake points contribute less to the average. This means the larger distances had very little impact but may add stability to the estimate. The other averages used a fixed distance and weighted all lake points within the fixed distance equally.

Finally, although small *p* values are commonly chosen to indicate statistical significance, it is also important to evaluate the size of the effects. The average of all estimated odds ratios for when PC is 100 μg/L is 1.476 (Fig. 5), meaning there is about a 48% increase in average ALS risk when the average PC exposure is 100 μg/L. This implies that locations, such as Missisquoi Bay, Lake Attitash, Lake Carmi, China Lake, or Showell Pond or any of the 32 lakes with PC >100 μg/L for example, have substantially elevated ALS risk.

The observed association between elevated PC levels and risk of ALS is supported by the implication of a water-related risk factor in our prior questionnaire-based case-control study in the region. Increased risk of ALS was associated with ever having lived full-time within 2 mi of a waterbody (OR 1.59 95%CI 1.05–2.42), and frequent participation in water sports, specifically boating, sailing, or kayaking (OR 1.51 95%CI 1.01–1.42), and particularly with water-skiing (adjusted OR 3.89 95%CI 1.97–8.44). Water-skiing retained independent statistical significance in a composite model containing age, gender, and smoking status (Andrew et al. 2017).

## Limitations

We note there are other potential risk factors for ALS and that some of these other risk factors potentially interact or reside in lakes that undergo CHABs. The array of environmental and occupational toxins that have been implicated include several, such as heavy metals lead and mercury (Kamel et al. 2005; Morahan and Pamphlett 2006; Kamel et al. 2008; Johnson and Atchison 2009), selenium (Vinceti et al. 1996), and agricultural pesticides (McGuire et al. 1997; Gunier et al. 2001), have all been proposed as influential drivers of sALS. Lifestyle factors and other toxins implicated also include tobacco (Nelson et al. 2000; Armon 2009), military service (Horner et al. 2008; Miranda et al. 2008), and head injuries (Chio et al. 2009; McKee et al. 2010; Lehman et al. 2012). We highlight that phycocyanin concentration is not necessarily representative of cyanotoxins or toxicity and more work is required to model these relationships. Furthermore, place of residence at time of diagnosis is not necessarily the location where etiologically relevant exposure occurred, and may not be representative of a person’s exposome (Jacquez et al. 2015; Sabel et al. 2009; Wheeler and Calder 2016). The temporal uncertainty may produce biased exposure estimates particularly if the water quality in this region was quite different at the time of onset of the disease in ALS cases compared to that at the relevant exposure time. Future work will continue to investigate spatial scales, toxins, and temporal uncertainty including chronic exposure, epigenetic factors, and lifetime residential history. Finally, although in this study we account for many of the model uncertainties, the PC proximity scales are all deterministic formulas of CHAB exposure that do not account for the uncertainty in the effect of the exposure (Waller and Gotway 2004). As technology provides more opportunities for direct measurements of cyanotoxins, these measurements can be further considered within a modeling framework.

## Conclusions

The ALS case dataset discussed in Caller et al. (2013) and Torbick et al. (2014) is further analyzed using measures of CHAB and robust spatial statistics that integrate uncertainty. We present the main conclusions in bulleted format to succinctly highlight the advances of this highly interdisciplinary research.

- We carried out extensive field data collection to calibrate and validate satellite remote sensing of cyanobacterial harmful algal bloom extent and intensity across northern New England. The mapping outcomes show robust PC mapping using Landsat ETM+ and OLI and in situ collected across multiple years, multiple path rows, seasons, and a range of lake conditions, indicating their usefulness for supporting public health investigations and CHAB tracking initiatives. This research provides the first regional estimates of PC exposure.
- This work builds upon previous efforts investigating the etiology of ALS and the potential role of lake water quality, CHAB, and BMAA toxins. This eco-epidemiological work used phycocyanin concentration, a direct measure of CHAB relative to previous works that used chlorophyll-a and other metrics (e.g., secchi depth, total nitrogen) of lake water quality.
- Our previous approach used a hot spot analysis which has potential limitations related to case ascertainment and/or potential challenges of modifiable area unit problems. This research does not explicitly use a hot spot approach and multiple scales of analysis were conducted.
- Robust and transparent spatial modeling used Bayesian inference of the Log-Gaussian Cox Process (LGCP), which includes spatial random effects specified by the logarithm of a Gaussian Markov Random Field on a regular lattice. For the estimation of the relationship between ALS risk and satellite-derived metrics phycocyanin concentration, the modeled component of the intensity for the LGCP, is defined by the water quality metrics as regression parameters combined with spatial random effects. This approach explicitly accounts for the spatial uncertainty of ALS “hotspots”.
- The spatial modeling shows that PC is found to be positively associated with ALS risk. However, the level of significance depends on several fixed components of the modeling framework. The effects of resolution choice of the regular lattice, the choice of background population, and the choice of spatial random effects (BYM versus none) were comprehensively investigated in terms of model fit by comparing the DIC values of different models. This work showed that the resolution choice had the largest impact on model fit, where the larger of the two resolutions gave the smallest DIC values. It is possible that the larger resolution provides a better model fit because population estimates in the rural regions are more stable at larger aggregation units and/or there is spatial uncertainty in potential exposure pathways.
- This research supports previous findings that poorer water quality are associated with higher likelihood of hot spot membership and the hypothesis that cyanotoxins are increasing the risk of ALS outcomes, and CHAB are a public health threat.
